# The role of food allergy‐related worry and self‐efficacy in explaining quality of life in caregivers of children

**DOI:** 10.1111/pai.70248

**Published:** 2025-11-17

**Authors:** Rebecca C. Knibb, Chloe Howell, Abbie Whitehouse, Catherine C. Peterson

**Affiliations:** ^1^ Aston Institute for Health and Neurodevelopment Aston University Birmingham UK; ^2^ School of Psychology Aston University Birmingham UK; ^3^ Atrium Health, Division of Pediatric Psychology and Neuropsychology Levine Children's Hospital Charlotte North Carolina USA; ^4^ School of Medicine Wake Forest University Charlotte North Carolina USA

**Keywords:** caregivers, food allergy, quality of life, self‐efficacy, worry

## Abstract

**Background:**

Food allergy (FA) is associated with considerable worry and poorer quality of life (QOL) in caregivers. High self‐efficacy for managing FA might reduce this impact but these relationships have not been examined with FA‐specific measures. This study aimed to explore relationships between worry, QoL and self‐efficacy using FA‐specific measures and provide further validation data for a revised Worry About Food Allergy (WAFA) scale, enabling differences in worry across child age to be examined.

**Methods:**

Caregivers of children with a food allergy (*n* = 240), recruited through patient organizations, completed the Worry About Food Allergy (WAFA) scale, Food Allergy Quality of Life Parental Burden Scale (FAQL‐PB), Food Allergy Self‐Efficacy scale for Parents (FASE‐P) and clinical and demographic questions.

**Results:**

The revised WAFA demonstrated excellent reliability for the full pre‐school, child and teen versions (Cronbach's alphas .95–.96) and short‐form versions (Cronbach's alpha .87–.92). Caregivers of 0‐5 year olds had significantly more FA‐related worry and less self‐efficacy for managing FA in social occasions than caregivers of 12–17 year olds (*p*s < .05); caregivers of 6–11 year olds reported poorer QoL than those of 12–17 year olds (*p* < .05). Caregiver‐reported FA severity, child age, greater worry and poorer self‐efficacy correlated with poorer QoL (all *p*s < .05–.01) and in regression models were significantly associated with QoL (all *p*s < .001), explaining 70% of the variance, with worry being the largest predictor.

**Conclusion:**

Caregivers of younger children may feel greater worry and burden of FA compared to caregivers of teenagers. Reducing FA‐worry and increasing FA‐self‐efficacy should be prime targets to improve FA‐QoL in caregivers.

**Key message**

Food allergy specific worry is an important determinant of quality of life in caregivers. Caregivers of younger children with food allergy may feel greater anxiety and burden compared to caregivers of teenagers. Reducing food allergy worry and increasing food allergy self‐efficacy should be prime targets to improve quality of life in caregivers. The newly revised Worry About Food Allergy scales are valid and reliable and could be useful as a screening tool in a clinical setting.

## 1 INTRODUCTION

Food allergy (FA) is a long‐term condition that is consistently associated with significantly reduced health‐related quality of life (HRQoL) amongst patients and caregivers.^1^, ^2^ For children and teens this seems to be particularly due to the social consequences of having FA and worries about having an accidental reaction outside of the home.^1^ A scoping review of caregiver burden of FA found that 83% of studies reported lower caregiver HRQoL in at least one area of caregivers' lives, particularly for caregivers of children with severe reaction history, and higher perceived severity ratings of their child's FA.^2^ Similar to children and teens, this stemmed in part from the persistent threat of their child being exposed to the allergen and the social and practical demands of managing FA.^2^

Having FA is also related to psychological distress, such as anxiety and depressive symptoms.^3^, ^4^, ^5^, ^6^ A recent global survey of nearly 4000 adults with FA and caregivers of children with FA found that nearly 80% reported some form of FA‐related distress, with anxiety of an allergic reaction being the most common.^7^ Anxiety related to FA has been reported by caregivers of a child with FA regardless of formal medical diagnosis,^3^ and the age of the child and history of severe reactions including anaphylaxis correlate with greater anxiety and parental burden outcomes.^4^

Understanding the determinants of FA‐related QoL and how this relates to FA‐related distress is important for directing clinical and supportive interventions for caregivers. Studies have shown that although clinical characteristics of FA such as the number of allergens, history of severe reaction or prescription of an adrenaline auto‐injector for the child can in part explain the burden on caregiver FA‐QoL, they may not be as important as psychological factors such as caregiver anxiety or confidence in FA managment.^8^ A caregiver's confidence in their abilities to manage their child's FA, or FA self‐efficacy has been reported to explain more variance in caregiver FA‐QoL than demographic variables, clinical factors or general health. Self‐efficacy for managing social activities and preventing allergic reactions in their child seems to be particularly important.^8^

Many studies looking at predictors of QoL in caregivers of children with FA have used generic measures, particularly for anxiety due to the lack of FA‐specific scales. This is beneficial for comparison to population norms, but generic measures lack sensitivity to FA‐specific concerns.^3^ Soller et al.^9^ compared a generic measure, the State–Trait Anxiety Inventory (STAI), to a FA‐specific visual analogue scale for anxiety and found that the STAI failed to capture over 30% of parents who reported high anxiety on the analogue scale. By exploring specific FA‐related dimensions using appropriate measures, treatment approaches derived from evidence‐based practice will be more beneficial.^9^ A FA‐related worry scale, Worry About Food Allergy (WAFA), has been recently developed in the US,^10^ but has not yet been used extensively in research. Worry relates to specific cognitive processes or thoughts about a potential problem or situation. The WAFA asks about the frequency of caregiver worries about their child's FA management and has been validated as a 13‐item scale on 265 caregivers of children with FA. It has good internal reliability with a Cronbach's alpha of .89 and displayed moderate convergent validity with generic measures of anxiety. It also exhibited small but significant correlations with clinical characteristics such as the number of allergens, number of symptoms, allergy reactions in the past 6 months, visits to the emergency room, hospitalisations, use of an adrenaline auto‐injector (AAI) and anaphylaxis.^10^ The authors have since revised the scale, using participant feedback from the original study of the 13‐item scale as well as input from an expert panel (e.g., allergy physicians, nurses, psychologists), to create three versions which can be used for parents of pre‐school‐aged children (ages 0–5), school‐aged children (ages 6–11). and teens (ages 12–17).

This study aims to build on previous research by using FA‐specific measures to further understand the determinants of caregiver FA‐QoL in order to provide direction for clinical care or for psychological interventions. Using the newly revised WAFA and FA‐specific scales to look at QoL and self‐efficacy, this study examines the relationships between these variables across different ages of children, alongside demographic and clinical characteristics. We also report on the psychometric properties of the newly revised WAFA scale.

## 2 METHODS

### 2.1 Design

The present study used a quantitative cross‐sectional design, using validated FA‐specific questionnaire measures assessing QoL, self‐efficacy and worry in caregivers. Ethical approval was obtained by the School of Psychology Research Ethics Committee at Aston University (REC ID: SP/2023/UG/01). All participants provided informed consent (online, prior to completing the online survey).

### 2.2 Participants and procedure

Participants were recruited as a convenience sample from the general population through advertisements distributed by national allergy charities: Anaphylaxis UK, Allergy UK, Natasha Allergy Research Foundation as well as social media platforms such as X (formerly known as Twitter) and Facebook. Participants had to be caregivers of at least one child (aged 0–17 years) with a medically or caregiver‐diagnosed FA and living in the UK. Caregiver‐diagnosed FA was included as research has shown that a medical diagnosis is not needed for caregivers to demonstrate FA‐related worry^3^ and poorer QoL.^11^ Particular age ranges of children were not targeted for recruitment, but numbers of caregivers of children of different age categories were monitored during the recruitment period. After giving online consent, participants completed the questionnaires online, hosted on the Qualtrics survey platform.

### 2.3 Measures

The Food Allergy Quality of Life‐Parental Burden Questionnaire (FAQL‐PB)^12^ is a 17‐item scale assessing FA‐specific QoL. This measure uses a 7‐point Likert scale ranging from 1 (not troubled) to 7 (extremely troubled) based on the level of burden related to the management of their child's FA. A total score can be used as well as two sub‐scales: Emotional Impact and Limitations on Life. A higher score on the FAQL‐PB indicates greater parental burden. Previous research has determined that the scale has high internal reliability (*α* > .85) and is valid for use in the UK.^13^ For the current study sample the FAQL‐PB had excellent internal consistency (overall *α* = .90; Limitations on Life *α* = .91; Emotional Impact *α* = .95).

Food Allergy Self‐Efficacy scale for Parents (FASE‐P)^14^ measures parental confidence in FA management using a 21‐item scale scored 0–100, with higher scores indicating higher confidence. This measure has an overall self‐efficacy score and has five subscales: prevention of an allergic reaction, managing social activities, identifying allergens, information seeking and treating an allergic reaction. This scale has good to excellent internal consistency across the subscales, as well as for the total measure (*α* = .88).^14^ For the current study sample it had satisfactory to excellent internal consistency (*α* = .96 for total scale; .62–.89 for the subscales).

Worry about Food Allergy Questionnaire (WAFA)^10^ is a FA‐specific worry unidimensional questionnaire. The original 13‐item scale has been further developed into three versions dependent on child age: WAFA‐P for pre‐school (0–5 years), WAFA‐C for children (6–11 years) and WAFA‐T for teens (12–17 years). Full scales range from 22 to 27 items and short forms of each have been proposed by the measure authors, ranging from 7 to 10 items. Caregivers rate their worry about their child's food allergy on a 5‐point Likert scale from 0 = Never to 4 = Every day. Sample items include “How often did you become nervous or worried about your child having a food allergy reaction?” and higher scores on the WAFA indicate greater worry. The initial WAFA scale had good internal consistency (α = .89) and moderate convergent validity with generic anxiety measures.^10^ Psychometric properties for the revised WAFA can be found in the results section. Scale items can be found in Appendix S1.

Participants completed a questionnaire to obtain demographic information as well as FA‐related information concerning their child. Questions gathered data concerning their child's type of FA, symptoms, diagnosis, history of anaphylaxis, and hospital admission.

### 2.4 Data analysis

Data was analyzed using SPSS (vn29), and all tests were 2‐tailed apart from t‐tests on clinical characteristics which were 1‐tailed, as it was expected that indicators of severity would relate to poorer QoL. The significance level was set at *p* < .05. Tests for normality, skew and kurtosis were acceptable (within −1 to +1 for skew and kurtosis, apart from FASE‐P sub‐scales prevention of an allergic reaction with kurtosis of 2.5 and identifying allergens with a kurtosis of 3.3 which is moderately acceptable).^15^ Cronbach's alpha was run to explore the internal reliability of the WAFA scales. Mean scores for all scales were then computed for further analysis. For the WAFA, mean scores were calculated for the P, C, and T versions of the scale and a mean score was also calculated for the whole sample. Pearson's bivariate correlations were conducted to explore relationships between FA‐related QoL, worry and self‐efficacy and continuous variables such as age. Independent *t*‐tests and ANOVAs examined differences in scale scores across age categories and differences in QoL across demographic variables including AAI prescription, history of anaphylaxis and hospitalization due to FA. Post hoc (Tukey) comparisons were conducted on significant ANOVA results to determine significant differences in group means. Hierarchical linear regression models were run with QoL as the outcome, including only significant predictor variables (*p* < .05). Post hoc power calculations showed the study had 99% power to detect a medium effect size with alpha at .5 for correlations, 94% power for ANOVAs and 99% for linear regression models with up to 9 predictors.^16^

## 3 RESULTS

A total of 240 caregivers completed the questionnaires; *n* = 87 of children aged 0–5 years, *n* = 96 of children aged 6–11 years and *n* = 57 of children aged 12–17 years. Demographic and FA characteristics of respondents are as shown in Table 1. Almost all (90.8%) of caregivers reported a medical diagnosis of FA for their child and 89.6% of children had an adrenaline auto‐injector (AAI) prescription. The most commonly reported allergy was peanuts (reported by 61.7% of caregivers), and the most common symptoms reported were skin rash (82.9%). Most caregivers rated their child's FA as severe (75.4%) and 68.8% reported their child had been admitted to hospital due to their FA.

**TABLE 1:** Characteristics of respondents (*n* %).

	Sample *n* = 240 *n* (%)
Parental Age (mean, s.d)	41.1 (6.29)
Gender of parent	
Male	9 (3.8)
Female	230 (95.8)
Prefer not to say	1 (0.4)
Ethnicity	
White British	169 (70.4%)
Black, African, or Caribbean	1 (0.4)
Asian	9 (3.8)
Mixed ethnicity	7 (2.9)
Prefer not to say	3 (1.3)
Child age in years (mean, s.d.)	7.98 (4.5)
Diagnosis	
Clinical history only	10 (16.3)
Skin prick test	167 (69.6)
Blood test	130 (54.2)
Food challenge	60 (25.0)
Parent diagnosed only	7 (2.9)
Foods reported	
Cereals (wheat, rye, barley, oats)	23 (9.6)
Crustaceans	17 (7.1)
Eggs	102 (42.5)
Fish	18 (7.5)
Milk	97 (40.4)
Tree Nut	113 (47.1)
Peanut	148 (61.7)
Sesame	64 (26.7)
Soya	18 (7.5)
Fruit	37 (15.4)
Vegetables	32 (13.3)
Other	77 (32.2)
Symptoms	
Wheezing	135 (56.3)
Tight Chest	94 (39.2)
Breathless	103 (46.0)
Rash	199 (82.9)
Itchy skin	131 (54.6)
Tingling in mouth, throat or ear	116 (48.3)
Swelling of face	159 (66.3)
Swelling of throat	100 (41.7)
Vomiting	130 (54.2)
Abdominal pain	108 (45.0)
Anaphylaxis	137 (57.1)
Hospital admission	165 (68.8)
Ambulance called due to reaction	133 (55.4)
AAI prescription	215 (89.6)
Been given an AAI injection	88 (36.7)
Parental severity rating	
Mild	8 (3.3)
Moderate	51 (21.3)
Severe	181 (75.4)

*Note*: Where % total more than 100, caregivers were able to select more than one answer. Where % total less than 100, caregivers did not provide an answer.

### 3.1 Psychometric properties of the revised WAFA scale

The full and short versions of the WAFA demonstrated excellent internal consistency. Cronbach's alphas for the WAFA‐C pre‐school (22 items), WAFA‐C child (24 items), and WAFA‐T teen (27 items) full scales were .95, .95, and .96, respectively. Cronbach's alphas for the pre‐school (10 items), child (10 items) and teen (7 items) short form scale were .92, .89, and .87, respectively. Full versions of the WAFA had strong significant correlations with QoL and moderate significant correlations with total self‐efficacy, with greater worry relating to poorer QoL and self‐efficacy. In particular, worry as measured by all age‐related forms for the WAFA correlated moderately to strongly with lower self‐efficacy for managing social occasions (see Table 2). Greater worry also significantly correlated with parent‐rated severity of FA (apart from the teen version) and the WAFA score combined across all ages significantly correlated with child age and caregiver age (Table 2).

**TABLE 2:** Relationship (Pearson's *r*) between quality of life, worry, self‐efficacy, and demographic variables.

Scale	FAQL‐PB	WAFA‐P	WAFA‐C	WAFA‐T	WAFA
		0–5 years	6–11 years	12–17 years	All ages
WAFA (all ages)	.77**	–	–	–	–
WAFA‐C (0–5 years)	.79**	–	–	–	–
WAFA‐P (6–11 years)	.75**	–	–	–	–
WAFA‐T (12–17 years)	.78**	–	–	–	–
FASE‐P (total)	−.56**	−.39**	−.35**	−.49**	−.41**
FASE‐P subscales					
Precaution	−.51**	−.33**	−.40**	−.29	−.36**
Reaction	−.18*	−.08	.03	−.31*	−.09
Identification	−.19*	−.14	−.16	−.04	−.15*
Seeking Information	−.33**	−.13	−.24*	−.26	−.21**
Social Activities	−.62**	−.48**	−.41**	−.57**	−.49**
Child Age	−.19**	−.25*	−.02	−.25	−.23**
Caregiver age	−.12	−.10	−.15	−.23	−.21**
Parental severity rating	.43**	.40**	.23*	.13	.25**

* *p* < .05.

** *p* < .01.

### 3.2 Relationships between QoL, worry, and self‐efficacy in caregivers

Better QoL was significantly correlated with less worry for the whole sample and for all three child age categories (*p* < .001). Better QoL was also significantly correlated with better self‐efficacy overall, and for each area of self‐efficacy measured by the FASE‐P (*p* < .001) (Table 2).

### 3.3 Differences in worry, self‐efficacy, and QoL for caregivers across ages for children with FA

Mean scores for all scales can be found in Table 3. There were significant differences in caregiver worry about food allergy across ages of child (*F*(2, 239) = 4.30, *p* = .01), with worry of caregivers of children aged 0–5 years being significantly higher than that of caregivers of children aged 12–17 years (post hoc *p* = .01).

**TABLE 3:** Means (and standard deviations) of caregiver QoL, self‐efficacy and worry about food allergy, split by child age.

Scale	Mean (SD) for whole sample	Mean (SD) for 0–5 years	Mean (SD) for 6–11 years	Mean (SD) for 12–17 years
Quality of life (FAQL‐PB)	5.00 (1.37)	5.07 (1.48)	5.20 (1.27)	4.58 (1.31)
Emotional Impact	5.21 (1.43)	5.28 (1.56)	5.43 (1.31)	4.73 (1.35)
Limitations on Life	4.62 (1.42)	4.69 (1.49)	4.77 (1.38)	4.31 (1.38)
FA Self‐Efficacy (FASE‐P)	69.95 (14.85)	67.44 (14.63)	69.72 (15.69)	73.70 (13.17)
Managing social activities	63.19 (22.69)	57.21 (23.76)	63.13 (21.04)	71.54 (21.75)
Precaution and prevention	72.20 (17.64)	69.80 (17.89)	72.87 (17.63)	74.38 (17.33)
Allergic reaction treatment	82.48 (18.93)	80.95 (19.81)	83.93 (18.40)	82.13 (18.78)
Food allergen identification	84.35 (15.91)	83.02 (16.88)	84.28 (15.81)	86.34 (14.76)
Seeking information	60.36 (19.57)	59.02 (17.88)	59.90 (21.30)	62.93 (18.87)
Worry about FA (WAFA)	1.99 (0.95)	2.18 (1.00)	1.98 (0.92)	1.71 (0.87)

There were also significant differences in QoL across ages of child (*F*(2, 224) = 3.78, *p* < .05), with QoL being significantly lower for caregivers of children aged 6–11 years compared to caregivers of children aged 12–17 years (post hoc *p* < .05). This was also the case for the emotional impact sub‐scale (*F*(2, 1224) = 4.48, *p* = .01; post hoc *p* = .01).

There were no significant differences in overall self‐efficacy across child age, but there were for self‐efficacy for managing social situations (*F*(2, 183) = 5.45, *p* < .05). Caregivers of children aged 0–5 years had significantly lower self‐efficacy than those with children aged 12–17 years (post hoc *p* < .01).

### 3.4 Relationships between QoL and clinical and demographic characteristics

Caregivers reported significantly lower QoL if their child had an adrenaline auto‐injector (AAI) prescription, had used an AAI in the past, had an ambulance called for them due to their FA, had been to the emergency room due to their FA, had anaphylaxis or reported their child's FA as severe (Tables 2 and 4). Poorer caregiver QoL was significantly related to the younger age of the child (Table 2), but QoL was not related to the age of the caregiver. No significant differences in QoL were found regarding the caregiver's relationship to the child, ethnicity, or being a support group member.

**TABLE 4:** Means (and standard deviations) of caregiver QoL across different clinical characteristics.

Clinical characteristic	Mean (SD)	Mean (SD)	*t*‐test (df)	*p* Value
	Yes	No		
AAI prescription	5.12 (1.23)	3.89 (1.88)	2.92 (21.89)	<.001
Use of AAI	5.42 (1.24)	4.89 (1.29)	2.98 (208)	<.01
Had to call an ambulance	5.39 (1.20)	4.51 (1.41)	4.96 (190.12)	<.001
Attendance at emergency room	5.23 (1.30)	4.48 (1.35)	3.93 (220)	<.001
Anaphylaxis	5.10 (1.33)	4.63 (1.59)	1.93 (212)	<.05

### 3.5 Associations with quality of life in caregivers after controlling for clinical and demographic variables

Variables that had a significant relationship with QoL were entered into a hierarchical linear regression model (Table 5). Clinical and demographic characteristics were entered in step 1, perceived severity of FA at step 2, self‐efficacy at step 3 and worry at step 4, in order to control for and explore the additional variance explained by each variable. All models were statistically significant with the final model (*F*(9, 173) = 49.77, *p* < .001) explaining 72% of the variance in QoL (*R* ^2^ = 0.73; Adj *R* ^2^ = 0.72). In step one, child age and prescription of an AAI were the only significant variables, with younger child age and having an AAI prescription associating with poorer QoL. In step two, child age remained significant alongside the severity of allergy, with greater severity associating with poorer QoL. AAI prescription was no longer significant. In step 3, child age, severity and self‐efficacy were significant (with greater self‐efficacy relating to better QoL), but in step 4 with the addition of worry, only severity, self‐efficacy and worry remained significant, with worry having the strongest association with QoL (see Table 5).

**TABLE 5:** Hierarchical linear regression to explore associations with quality of life.

Predictors	Unstandardised B	Standardized B	*t*	95% CIs	
				Lower	Upper
Step 1					
Child age	−0.07	−0.23**	−3.22	−0.12	−0.03
AAI prescription	−0.96	−0.21**	−2.56	−1.71	−0.22
Use of AAI	−0.28	−0.12	−1.24	−0.72	0.17
Need to call ambulance	−0.43	−0.16	−1.78	−0.90	0.05
Been to hospital	−0.14	−0.05	−0.61	−0.61	0.32
Anaphylaxis	0.05	0.02	0.24	−0.34	0.43
*R* ^2^; Adj *R* ^2^	0.19; 0.17				
*F* value; *F* change	6.70***; 6.70***				
Step 2					
Child age	−0.09	−0.28***	−4.07	−0.13	−0.04
AAI prescription	−0.42	−0.09	−1.09	−0.70	0.20
Use of AAI	−1.8	−0.08	−.84	−0.60	0.24
Need to call ambulance	−0.25	−0.10	−1.09	−0.70	0.20
Been to hospital	−0.05	−0.02	−0.21	−0.48	0.39
Anaphylaxis	0.15	0.06	0.80	−0.22	0.51
Severity rating	1.03	0.39***	4.87	0.61	1.45
*R* ^2^; Adj *R* ^2^	0.30; 0.27				
*F* value; *F* change	9.91***; 23.71***				
Step 3					
Child age	−0.06	−0.20***	−3.33	−0.10	−0.03
AAI prescription	−0.39	−0.09	−1.2	−1.02	0.24
Use of AAI	−0.13	−0.05	−0.69	−0.48	0.23
Need to call ambulance	−0.15	−0.06	−0.77	−0.54	0.24
Been to hospital	−0.12	−0.04	−0.62	−0.49	0.26
Anaphylaxis	0.18	0.07	1.12	−0.14	0.49
Severity rating	0.82	0.31***	4.45	0.46	1.18
Self‐efficacy	−0.04	−0.45***	−7.75	−0.05	−0.03
*R* ^2^; Adj *R* ^2^	0.48; 0.46				
*F* value; *F* change	19.23***; 60.03***				
Step 4					
Child age	−0.02	−0.06	−1.35	−0.05	0.01
AAI prescription	−0.24	−0.05	−1.04	−0.69	0.22
Use of AAI	−0.05	−0.02	−0.37	−0.31	0.21
Need to call ambulance	−0.14	−0.05	−0.95	−0.42	0.15
Been to hospital	−0.03	−0.01	−0.21	−0.30	0.24
Anaphylaxis	0.19	0.07	1.63	−0.04	0.41
Severity rating	0.57	0.22***	4.22	0.30	0.83
Self‐efficacy	−0.02	−0.27***	−5.93	−0.03	−0.02
Worry	0.83	0.58***	12.35	0.70	0.97
*R* ^2^; Adj *R* ^2^	0.73; 0.72				
*F* value; *F* change	49.77***; 152.45***				

** *p* < .01.

*** *p* < .001.

## 4 DISCUSSION

The aim of the present research was to gain a greater understanding of the relationships between clinical and demographic variables and FA‐related QoL, worry and self‐efficacy in caregivers of children with FA, using FA‐specific measures. This study has provided novel findings on the validity of the revised WAFA scale and the relationships between QoL, worry and self‐efficacy and potential risk factors for determining which parents may need support in managing their child's FA. In our sample of participants, the WAFA scales demonstrated excellent reliability for each age version, both for the long and short forms. The WAFA scales also performed as expected in relation to construct validity, with moderate to strong significant correlations in the expected directions with self‐efficacy, QoL and FA severity. These scales provide researchers and clinicians with options to measure caregiver worry specific to the age of the child, with the short forms being of particular benefit for use in a busy allergy clinic.

Similar to previous work using generic anxiety scales,^17^ significant associations were found between higher caregiver allergy‐related worry and poorer QoL. Poorer self‐efficacy for FA management was also related to poorer QoL for caregivers. Although we found this association in all age groups, the more nuanced analysis showed that caregivers of 0–5 year olds had significantly more FA‐related worry and less self‐efficacy for managing FA in social occasions than caregivers of 12–17 year olds. Caregivers of children aged 6–11 years reported poorer QoL than those of children aged 12–17 years. It may be that for younger children, caregivers have not had the chance to develop high levels of confidence in managing FA for their child and are more worried about the consequences of mismanagement, particularly as situations involving food continuously evolve and change for the younger child as they become more able to take part in social activities. Older children also take on more of the responsibility of their FA themselves, relieving some of the burden from the caregiver.^18^ It is also possible the association here relates to the length of time since diagnosis and how long a caregiver has had to develop management skills. Time since diagnosis should be investigated in future studies.

Similar to previous studies, caregivers reported significantly lower QoL if their child had more severe FA,^2^, ^17^ indicated by prescription or use of an AAI, calling an ambulance or attending an emergency room, had anaphylaxis or reported their child's FA as severe. It is understandable that these factors may lead to caregivers restricting social activities involving food and greater concern about their child having another reaction. However, once self‐efficacy and worry were added into regression models, only perceived severity retained a significant association, with greater perceived severity, poorer self‐efficacy for FA management and greater FA‐related worry, relating to poorer caregiver QoL. This has been found in previous research, where clinical variables became less important once psychological variables such as self‐efficacy were added into models.^8^ Caregiver‐perceived severity for FA has also been reported to be a strong and significant predictor of FA‐related QoL even after controlling for other variables.^17^, ^19^ These findings are encouraging, as it appears that factors most important in explaining QoL are modifiable and can be targeted within clinical settings or behavior change interventions.

FA‐related worry had the strongest association with, and appears to be one of the most important determinants of, QoL. The perceived risk of the severity of their child's FA and risk of a potentially fatal reaction can lead to heightened vigilance behaviors in parents and restriction of activities involving food, which can exacerbate worry. Avoidance of activities that are seen as stressful or anxiety provoking means that there is no opportunity to learn how to manage these activities, further increasing worry about them and reducing self‐efficacy and QoL. Findings from this study suggest that this interplay between worry, self‐efficacy and QoL may be particularly the case for younger children, where parents are learning how to manage their child's FA as their child grows, alongside opportunities for them to be in contact with an allergen. The cross‐sectional nature of this study does need to be considered when interpreting these findings though. It could equally be the case that poorer QoL due to the impact of severe FA and lower self‐efficacy for managing the condition could lead to much greater worry about the impact FA is having, and the caregiver's abilities to keep their child safe across different scenarios.

All domains of self‐efficacy were associated with QoL in this study; however, self‐efficacy for navigating social situations was found to be associated most strongly with QoL and related to caregiver worry. It was also poorest in caregivers of 0–5‐year‐olds compared to older children. This facet of self‐efficacy has been strongly associated with caregiver QoL in previous research.^8^ Support for caregivers in managing FA in social situations, particularly for those of younger children, may therefore be advantageous, in order to improve QoL and reduce worry. Older children may begin to take on more of these responsibilities themselves, thus reducing the burden on the parent.^18^

There are limitations that should be acknowledged when considering the implications of the findings from this research. Due to the cross‐sectional nature of this study, it is not possible to establish causation and longitudinal research is needed to explore these relationships further so that predictive models can be developed in order to ensure support is directed at those most in need. This study sample was recruited mostly through national allergy associations and so it is possible that those most interested in or affected by the psychosocial issues related to FA participated in the study. Our sample was also predominantly comprised of white British females who were well educated, and so we are not able to say if these findings would be the same in male caregivers or in caregivers from different ethnicities or other socio‐economic groups. Similar findings regarding the association between food allergy self‐efficacy and QoL have been reported as existing in participants of different racial backgrounds,^20^ however further research in under‐represented groups is still needed in order to explore the robustness of these findings across such groups and ensure support is provided in the most optimal way. Such groups could be recruited through reaching out to local communities and offering study materials in different languages.^21^

Nevertheless, the study provides important direction for clinical care and support. An understanding of the level of caregiver worry about FA is extremely important. The revised WAFA scales may be a useful tool in a clinical environment to help identify those in need. Further, the short WAFA forms may be very useful as a screening tool in clinic, where time is limited. Measuring self‐efficacy may also provide clinicians with an understanding of the areas of FA management caregivers are struggling with. Caregivers of younger children and those who perceive their child to have a severe FA may need particular attention. The strengths of the relationships found in the present study also demonstrate the importance of using condition‐specific rather than generic measures.^3^

## AUTHOR CONTRIBUTIONS

**Rebecca C. Knibb:** Conceptualization; investigation; writing – original draft; methodology; writing – review and editing; formal analysis; project administration; data curation; supervision; resources. **Chloe Howell:** Investigation; writing – original draft; methodology; writing – review and editing; formal analysis. **Abbie Whitehouse:** Investigation; writing – original draft; methodology; writing – review and editing; formal analysis. **Catherine C. Peterson:** Conceptualization; writing – review and editing.

## FUNDING INFORMATION

No funds have been received for this study.

## CONFLICT OF INTEREST STATEMENT

RCK: research funding from the National Institute for Health Research, Aimmune, National Peanut Board, Novartis and the Food Standards Agency and honoraria from Nutricia, Viatris and DBV Technologies. RCK is also Chair of the British Society for Allergy and Clinical Immunology Psychology Special Interest Group for Psychology.

## PEER REVIEW

The peer review history for this article is available at https://www.webofscience.com/api/gateway/wos/peer‐review/10.1111/pai.70248.

## Supporting information


Appendix S1.
