# Supplementary material for: The role of food allergy‐related worry and self‐efficacy in explaining quality of life in caregivers of children
Source: Pediatr Allergy Immunol. 2025 Nov 17;36(11):e70248. doi: 10.1111/pai.70248 (PMC12621170; doi:10.1111/pai.70248)
Supplement: Supplementary file 1 — Appendix S1. [file PAI-36-e70248-s001.docx]

**The role of food allergy-related worry and self-efficacy in explaining quality-of-life in caregivers of children.**

**Supplementary material - Worry About Food Allergy scales**

**Worry about Food Allergy Parent-Report Preschool Version (WAFA-P-Preschool)**

Thinking about the past month, please select from the following options to indicate how often you have worried or become nervous about each item.

0 = Never

1 = Once a month

2 = Once a week

3 = A few times a week

4 = Every day

In the last month, how often did you become nervous or worried about:

…the possibility of your child having a food allergy reaction?

…carrying your child’s emergency medication?

…your child not receiving emergency medicine to treat a food allergy reaction when needed?

…your child dying because of a food allergy reaction?

…what others will think of your child when they have a food allergy reaction?

…pain your child might experience from using an epinephrine auto-injector?

…possible side effects of your child’s epinephrine auto-injector?

…your child experiencing the administration of self-injectable epinephrine as traumatic?

…your family having to miss out on social activities because of your child’s food allergy?

…your child having to miss out on social activities because of their food allergy?

…your child feeling different from other kids because of their food allergy?

…other adults/caregivers (e.g., sitters, family members, teachers, etc.) helping your child when they have a food allergy reaction?

…having only safe food offered by other adults/caregivers?

…your child becoming allergic to additional foods?

…your child trying new foods?

…your child’s food allergy symptoms or reactions getting worse?

…others understanding when you explain your child’s food allergy?

…your child being bullied or teased about their food allergy?

…your child having a food allergy reaction while traveling?

…your child having to undergo medical procedures (e.g., skin prick testing, oral food challenges)?

…the financial cost of allergy management (e.g., cost of epinephrine auto-injector, safe foods, medical testing, etc.)?

…recognizing if your child is having a food allergy reaction?

**Worry about Food Allergy Parent-Report Child Version (WAFA-P-Child)**

Thinking about the past month, please select from the following options to indicate how often you have worried or become nervous about each item.

0 = Never

1 = Once a month

2 = Once a week

3 = A few times a week

4 = Every day

In the last month, how often did you become nervous or worried about:

…the possibility of your child having a food allergy reaction?

…carrying or having your child carry their emergency medication?

…your child not receiving emergency medicine to treat a food allergy reaction when needed?

…your child dying because of a food allergy reaction?

…what others will think of your child when they have a food allergy reaction?

…pain your child might experience from using an epinephrine auto-injector?

…possible side effects of your child’s epinephrine auto-injector?

…your child experiencing the administration of self-injectable epinephrine as traumatic?

…your child missing school activities because of their food allergy?

…your family having to miss out on social activities because of your child’s food allergy?

…your child having to miss out on social activities because of their food allergy?

…your child feeling different from other kids because of their food allergy?

…your child being able to manage a food allergy reaction independently (e.g., self- administer epinephrine auto-injector or seek help from others to manage a food allergy reaction)?

…your child making safe food choices without your help (e.g., if they were with friends or at school and you weren’t there)?

…your child becoming allergic to additional foods?

…your child trying new foods?

…your child’s food allergy symptoms or reactions getting worse?

…your child needing to explain food allergy to other people without your help?

…others understanding when you explain your child’s food allergy?

…your child being bullied or teased about their food allergy?

…your child having a food allergy reaction while traveling?

…your child having to undergo medical procedures (e.g., skin prick testing, oral food challenges)?

…the financial cost of allergy management (e.g., cost of epinephrine auto-injector, safe foods, medical testing, etc.)?

…your child being able to accurately assess their risk of food allergy on their own?

**Worry about Food Allergy Parent-Report Teen Version (WAFA-P-Teen)**

Thinking about the past month, please select from the following options to indicate how often you have worried or become nervous about each item.

0 = Never

1 = Once a month

2 = Once a week

3 = A few times a week

4 = Every day

In the last month, how often did you become nervous or worried about:

…the possibility of your teen having a food allergy reaction?

…your teen carrying their emergency medication?

…your teen not receiving emergency medicine to treat a food allergy reaction when needed?

…your teen dying because of a food allergy reaction?

…what others will think of your teen when they have a food allergy reaction?

…pain your teen might experience from using an epinephrine auto-injector?

…possible side effects of your teen’s epinephrine auto-injector?

…your teen experiencing the administration of self-injectable epinephrine as traumatic?

…your teen missing school activities because of their food allergy?

…your family having to miss out on social activities because of your teen’s food allergy?

…your teen having to miss out on social activities because of their food allergy?

…your teen feeling different from other teens because of their food allergy?

…your teen being able to manage a food allergy reaction independently (e.g., self-administer epinephrine auto-injector or seek help from others to manage an allergic reaction)?

…your teen making safe food choices without your help (e.g., if they were with friends or at school and you weren’t there)?

…your teen becoming allergic to additional foods?

…your teen trying new foods?

…your teen’s food allergy symptoms or reactions getting worse?

…your teen needing to explain food allergy to other people without your help?

…others understanding when you explain your teen’s food allergy?

…your teen being bullied or teased about their food allergy?

…your teen having a food allergy reaction while traveling?

…your teen having to undergo medical procedures (e.g., skin prick testing, oral food challenges)?

…the financial cost of allergy management (e.g., cost of epinephrine auto-injector, safe foods, medical testing, etc.)?

…your teen’s risk of a food allergy reaction if they experiment with alcohol or drugs?

…your teen’s risk of a food allergy reaction from kissing someone or other intimate physical contact?

…your teen missing extracurricular activities or sports because of their food allergy?

…your teen being able to accurately assess their risk of food allergy on their own?

**Worry About Food Allergy scales – short forms**

**Worry about Food Allergy Parent-Report Preschool Version (WAFA-P-Preschool)**

Thinking about the past month, please select from the following options to indicate how often you have worried or become nervous about each item.

0 = Never

1 = Once a month

2 = Once a week

3 = A few times a week

4 = Every day

In the last month, how often did you become nervous or worried about:

…the possibility of your child having a food allergy reaction?

…carrying your child’s emergency medication?

…your child feeling different from other kids because of their food allergy?

…other adults/caregivers (e.g., sitters, family members, teachers, etc.) helping your child when they have a food allergy reaction?

…having only safe food offered by other adults/caregivers?

…your child trying new foods?

…your child’s food allergy symptoms or reactions getting worse?

…others understanding when you explain your child’s food allergy?

…your child having a food allergy reaction while traveling?

…recognizing if your child is having a food allergy reaction?

**Worry about Food Allergy Parent-Report Child Version (WAFA-P-Child)**

Thinking about the past month, please select from the following options to indicate how often you have worried or become nervous about each item.

0 = Never

1 = Once a month

2 = Once a week

3 = A few times a week

4 = Every day

In the last month, how often did you become nervous or worried about:

…the possibility of your child having a food allergy reaction?

…carrying or having your child carry their emergency medication?

…your child not receiving emergency medicine to treat a food allergy reaction when needed?

…your child feeling different from other kids because of their food allergy?

…your child being able to manage a food allergy reaction independently (e.g., self- administer epinephrine auto-injector or seek help from others to manage a food allergy reaction)?

…your child making safe food choices without your help (e.g., if they were with friends or at school and you weren’t there)?

…your child trying new foods?

…others understanding when you explain your child’s food allergy?

…your child having a food allergy reaction while traveling?

…your child being able to accurately assess their risk of food allergy on their own?

**Worry about Food Allergy Parent-Report Teen Version (WAFA-P-Teen)**

Thinking about the past month, please select from the following options to indicate how often you have worried or become nervous about each item.

0 = Never

1 = Once a month

2 = Once a week

3 = A few times a week

4 = Every day

In the last month, how often did you become nervous or worried about:

…the possibility of your teen having a food allergy reaction?

…your teen carrying their emergency medication?

…your teen not receiving emergency medicine to treat a food allergy reaction when needed?

…your teen feeling different from other teens because of their food allergy?

…your teen being able to manage a food allergy reaction independently (e.g., self-administer epinephrine auto-injector or seek help from others to manage an allergic reaction)?

…your teen having a food allergy reaction while traveling?

…your teen being able to accurately assess their risk of food allergy on their own?
